# Strictures on Dr. Kinglake's Paper on the Position of Fractured Limbs

**Published:** 1821-01

**Authors:** Charles W. Smerdon

**Affiliations:** Surgeon.


### *Strictures on* Dr. Kinglake's *Paper on the Position of Fractured Limbs.* By Charles W. Smerdon, Esq. Surgeon.

I SHOULD not have expected to witness a physician of respectability coming forward as an advocate of a practice in surgery so nearly exploded (at least in this country), as that of the straight position of fractured limbs. Such conduct in many men might very well be passed over in silence; but the talents of Dr. Kinglake are such as make it appear to me to be a point of professional duty in those who reprobate that practice, to take into consideration the arguments he advances on this occasion. Before, however, I adduce the strictures I have to make on them, I cannot neglect to remark, that Dr. Kinglake does not seem to be well acquainted with the character of the professional acquirements of British practitioners, or he would not have gravely asserted that they "sometimes do irreparable mischief by the absurd practice of aiming at salutarily stimulating the surfaces of the broken ends (of bones) by moving them on each other." Without fear of contradiction, I will answer for every surgeon who has received a regular education, that such a glaringly "absurd practice" was never conceived by him to be a measure at all plausible.

Dr. Kinglake sets out by saying, that "the earliest periods of practical surgery must have been those in which theoretical considerations could have but little influence." Theoretical considerations! What, is that still theoretical, which Pott wrote upon and advocated after a practice of more than thirty years, and which has since been sanctioned and followed by all the first surgeons in London?* Again he says, "to have insisted at that time that laying a broken bone in a curved position, would be favourable both to the osseous union and to the eventful straightness of the limb, would have been thought unaccountably strange, and altogether untenable." Dr. Kinglake has unintentionally written this sentence very obscure. Certainly, to place a broken bone in a curved position, is not the way to make it straight. In several places he has very improperly made use of the word curved instead of bent. Now, a curve, in the common acceptation of the word, signifies a part of the circumference of a circle; but, when a joint is bent, it forms an angle more or less acute, but certainly not a curve. But, to drop this sort of criticism, I will contend, that it is absurd to suppose that the science of surgery is to be stopped in its march towards perfection, because our forefathers would have thought its present improvements "unaccountably strange and altogether untenable."

In advocating the bent position, it is not intended to assert that all the muscles of a fractured limb are placed in a state of relaxation; and it is puerile to suppose that so good an anatomist as Mr. Pott intended literally to convey such a meaning. All that can be meant is, that such muscles are placed in a relaxed state by the bent position as are most likely to draw, by their contractions, the fractured pieces of bone from each other, and which are usually placed in a relaxed state by every animal when asleep or in a state of rest:† these muscles are the flexors and the adductors. Suppose that an artist were to pourtray a sleeping beauty, would he place her with her back perfectly straight, and her limbs stretched out to their utmost extent, as

* Without intending to express any opinion on the merits of either mode of treatment, we may remark that Pott's practice is not universally followed by surgeons of the first rank in London. At one of the hospitals here, the straight position of the limb, in cases of fracture of the thigh-bone, is generally adopted by the whole of the attending surgeons.—Edit.

† It may be here worthy of remark, that, in birds of flight, the largest muscles are those flexors which bring the wings to the side, when they are stretched out in the act of flying. These muscles, when at rest, are always in a relaxed state, and undoubtedly require more perfect freedom from distention than their antagonists' muscles do. The same may be remarked in the human subject, between the deltoid, the spinati, and other muscles that raise the arm, and those which are intended to bring it again to the side. The one set, for their rest, require perfect relaxation; whilst the others receive the same degree of ease although they are almost constantly stretched out. In short, it is evident that some muscles naturally require, for their rest, a perfect state of relaxation; whilst the antagonists to them receive the same quantity of rest, although kept upon the stretch.

if they were pinioned? Certainly not? He would, I should think, gently curve the back, throw her arms across the breast, and bend the joints of the hips and knees. This position, I believe, is that of the most perfect rest,—that, too, which nature points out as the best adapted for restoring to the muscles that vigour which they may have lost by previous action. Now, is it not reasonable to place muscles in the most natural state of ease and comfort, which are to remain in a fixed position for at least four weeks? and can any other position be better adapted than that which man naturally throws himself into when his muscles require ease and rest? One of the characteristic properties of muscular fibre, is to contract on the application of stimuli. Now, we know that the stimulus of distention is a very powerful agent for this purpose, as we may observe in the action of the heart, the blood-vessels, and the bowels. We have undoubted evidence, likewise, that the voluntary muscles contract powerfully, and remain permanently rigid, when they are violently and suddenly stretched: hence arises the only difficulty which is met with in the reduction of dislocations,—a difficulty which it is sometimes impossible to overcome without the most powerful mechanical re-agents.

In all cases of fractures, the great object of the surgeon is to effect a quiescent state of the muscles of the broken limb; and for this purpose it is his duty, as much as lies in his power, to withdraw from them every stimulus of whatsoever kind that is likely to affect them. Now, if distention be a stimulus to muscular fibre, (which, I presume, no one can doubt,) does he not withdraw it by placing the muscles in a relaxed position? and does he not, by this means, contribute considerably towards the general indications?

The next best means to produce a permanently quiescent state of muscles, is to brace them up moderately tight with a suitable bandage. No one will doubt this who has seen the effect of tight bandaging in chronic rheumatism, where the muscles are principally affected. In cases of lumbago attended with pain and spasmodic twitchings of the muscles of the back, I have seen the greatest relief obtained by applying pads on the lumbar mass, bound on as tight as possible by means of a broad flannel swathe. Nor is the good thus derived evanescent: if the plan be accompanied with perfect rest in a horizontal position, a cure may speedily be expected, without the aid of any other medicines than what are indicated by the state of the bowels.

If we instance a case of fractured thigh-bone, and enumerate the muscles which are relaxed by the bent position, we shall find that these exceed very considerably in power those which are placed in opposition to them. The position here meant is a slight flexion of the hip and knee, by the placing of a pillow in the ham. First, the psoæ muscles and the iliacus internus, which are inserted into the trochanter minor; 2dly, the sartorius, the rectus, and the gracilis; 3dly, the pectinalis and the triceps adductor, which are inserted into the whole length of the linea aspera; and, 4thly, the long flexors of the leg, the biceps, the semi-membranosus, and semi-tendinosus, which are inserted into the head of the tibia and fibula. These are all muscles of considerable power and extent, and, it is evident, are placed in a relaxed state by the bent position. A view of the skeleton, however, must convince us that some of these muscles, by spasmodic action in the case we are supposing, are capable of doing more mischief than the others. Thus, the three heads of the triceps adductor, which, taken collectively, form a very large mass of muscle, are those which, I believe, tend more to occasion deformity in the uniting of a fractured femur than all the rest put together. Their origin and insertion, in the entire state of the bone, point out that they must act powerfully in bringing the thighs together, and crossing them, if required. When, however, the femur is fractured, the action of the adductor brevis will draw the broken end of the upper part of the bone from that of the lower part; while this, the inferior portion, having now no fixed position, will be powerfully drawn, by the lower fibres of the magnus and longus, upwards and inwards.*

Dr. Kinglake goes on to state, "the range of involuntary action given to the muscles connected with a fractured bone when in a relaxed state, is such as operates most injuriously on the stationary position to which it would be desirable constantly to confine the broken ends of the bone." Here, for reasons which I have before stated, I am entirely at issue with him. If distention be a powerful stimulus to muscular fibre, and if muscles are liable to involuntary action by being stretched, whether is it the more rational plan, in order to prevent that action, to relax them, or to place them in a state of distention? Besides, when the rigidity of the muscular fibres is taken off, a bandage can be placed on a fractured extremity with more cer-

* If it be a matter of much consequence to relax the triceps femoris in cases of fractured thigh bone, it is necessary to adopt that sort of position which will secure this point with the greatest possible ease to the patient. It may be questioned if the usual posture of placing the limb on the outside, is the best under all the circumstances of the case. Certainly it is not, if the pelvis be not brought round, as it were, to the limb, so that the patient shall lie completely on his side: otherwise the triceps would evidently be placed on the stretch, and the fracture, if an oblique one, be in great danger of being displaced. Perhaps, the best general position is to place the patient on his back; to keep the joints bent, by placing a pillow, well rolled up, in the ham; to rest the limb on the heel, supported properly, and well kept up; and to place the whole extremity so that it may incline a little inward towards its fellow.

tainty of confining the bone by pressing the relaxed and pulpy muscles close around it, than if those muscles retained their natural plumpness and resistance. But Dr. Kinglake seems to have fallen out with all the modern improvements in the treatment of fractured limbs: improvements which, I will venture to affirm, a very large majority of hospital surgeons in this and the sister kingdom are daily in the habit of acting upon.

The preference given to long splints which comprehend the joint above and below the fractured bone, must rank as a very considerable improvement in modern surgery, and which every one competent to judge on the subject, and who at the same time is unbiassed by either prejudice or a love of singularity, will, I believe, allow. In fractures of the thigh-bone, where the muscles are large and powerful, and their range of action of considerable extent, it is very doubtful if short splints be of any other service than that of keeping the patient's mind quiet by a strict adherence to ancient and well-known custom: but I will go a step further, and venture to affirm that they may be productive of harm where there is a disposition in the muscular fibre to spasm, by the very partial and unequal pressure which they must make on the long muscles of the limb and on the bone. Let us instance the vasti muscles, which have their origin from the trochanters and from the whole length of the bone, and are inserted into each side of the patella in the entire state of the thigh: when these muscles act, they extend the leg; but, if the femur be fractured, their contractions will draw the inferior extremity of the bone upwards, and thus increase the deformity. As a remedy for this evil, (as far, at least, as a remedy is within our power,) common sense, I should think, would point out the propriety of making an equable pressure throughout the whole length of such muscles: and where, from the shape of the limb or some other cause, this cannot be done by the splints alone, pads should be introduced to make up for the inequality.

In his treatment of compound fractures, "attended with much contusion, and even threatening laceration of the soft parts," Dr. Kinglake must have been more than ordinarily successful, if he has obtained effectual and speedy cures by "binding up the soft parts with sufficient strictness to keep the broken ends in contact." I agree that it should be the first wish of the surgeon to bring about a union of the external wound as soon as possible; and, where this can be done by Dr. Kinglake's plan, it is but fair to say that a better cannot be adopted. But, in cases where the contusion of the soft parts is very considerable, and where the loss of vitality of such parts may be seriously apprehended, as a consequence of the violent inflammation which generally follows contused and lacerated wounds, I apprehend that such a practice is very likely to produce the evil which it is intended to avoid. In such wounds of muscular parts the re-action which follows is always in the extreme, and the blood-vessels of the part become excited to the utmost degree, which will continue until nature relieves herself by an effusion into the cellular tissue, as is indicated by tumefaction and a comparative relief from pain. But how can such a salutary process be brought about if the affected muscles be bound up tight by a bandage? If it were necessary in order to deprecate a practice so evidently injudicious, I could here relate a case of this sort of wound, in which the most extensive sloughing was obviously produced by tightly bandaging the part, and by the application of cold evaporating lotions, with the view of bringing about union by the first intention, as it is called. In all such cases it is manifestly the duty of the surgeon to be rather the servant than the master of nature, and to assist her in her salutary operations by relaxing fomentations and local bleeding, rather than to impede her by the practice here pointed out.

*Clifton, Bristol; Oct.* 1820.

